# Extracellular vesicles from bone marrow mesenchymal stem/stromal cells transport tumor regulatory microRNA, proteins, and metabolites

**DOI:** 10.18632/oncotarget.3211

**Published:** 2014-12-31

**Authors:** Krishna C. Vallabhaneni, Patrice Penfornis, Santosh Dhule, Francois Guillonneau, Kristen V. Adams, Yin Yuan Mo, Rui Xu, Yiming Liu, Kounosuke Watabe, Mohan C. Vemuri, Radhika Pochampally

**Affiliations:** ^1^ Cancer Institute, University of Mississippi Medical Center, Jackson, MS, USA; ^2^ Department of Chemical and Biomolecular Engineering, New Orleans, LA, USA; ^3^ 3P5 Proteomic Platform of the Université Paris Descartes, Sorbonne Paris Cité, Paris, France; ^4^ Department of Pathology, University of Mississippi Medical Center, Jackson, MS, USA; ^5^ Department of Chemistry and Biochemistry, Jackson State University, Jackson, MS, USA; ^6^ Stem Cell Biology, Thermo Fisher Scientific, Frederick, MD, USA; ^7^ Department of Biochemistry, University of Mississippi Medical Center, Jackson, MS, USA

**Keywords:** Mesenchymal Stem Cells, microRNA, breast cancer, exosomes, tumor microenvironment

## Abstract

Human mesenchymal stem/stromal cells (hMSCs) have been shown to support breast cancer cell proliferation and metastasis, partly through their secretome. hMSCs have a remarkable ability to survive for long periods under stress, and their secretome is tumor supportive. In this study, we have characterized the cargo of extracellular vesicular (EV) fraction (that is in the size range of 40-150nm) of serum deprived hMSCs (SD-MSCs). Next Generation Sequencing assays were used to identify small RNA secreted in the EVs, which indicated presence of tumor supportive miRNA. Further assays demonstrated the role of miRNA-21 and 34a as tumor supportive miRNAs. Next, proteomic assays revealed the presence of ≈150 different proteins, most of which are known tumor supportive factors such as PDGFR-β, TIMP-1, and TIMP-2. Lipidomic assays verified presence of bioactive lipids such as sphingomyelin. Furthermore, metabolite assays identified the presence of lactic acid and glutamic acid in EVs. The co-injection xenograft assays using MCF-7 breast cancer cells demonstrated the tumor supportive function of these EVs. To our knowledge this is the first comprehensive -omics based study that characterized the complex cargo of extracellular vesicles secreted by hMSCs and their role in supporting breast cancers.

## INTRODUCTION

Human mesenchymal stem/stromal cells (hMSCs) are plastic adherent cells derived from bone marrow, referred commonly in the hematological literature as marrow stromal cells and later classified as multipotent mesenchymal stromal cells [1]. Various studies have shown that hMSCs act as stromal cells for solid tumors where they localize, integrate into the tumor associated stroma [2-5]. Once integrated, apart from providing stromal support, hMSCs promote tumor growth and angiogenesis [6, 7] through juxtacrine, paracrine and endocrine mechanisms [2, 8]. However the underlying mechanism by which hMSCs support tumor growth remains largely unexplored.

Previously, our lab established an *in vitro* model system to study stromal cell survival under conditions that mimic the nutrient deprived core of solid tumors [9, 10]. Serum deprived hMSCs (SD-MSCs) survive complete serum withdrawal using catabolic pathways such as autophagy, and they undergo specific epigenetic changes and secrete factors that support breast tumor survival and growth. Furthermore, we and others have shown that hMSCs secrete bioactive molecules such as IGF-1, VEGF, MMP proteins that act as paracrine mediators which either directly act on the target cells or stimulate the neighboring cells to secrete functionally active molecules that are known to inhibit apoptosis, enhance angiogenesis, and help in tissue regeneration [11-13]. In this study, we set out to complete the characterization of the extracellular vesicular (EV) fraction of SD-MSCs secretome.

**Figure 1:**
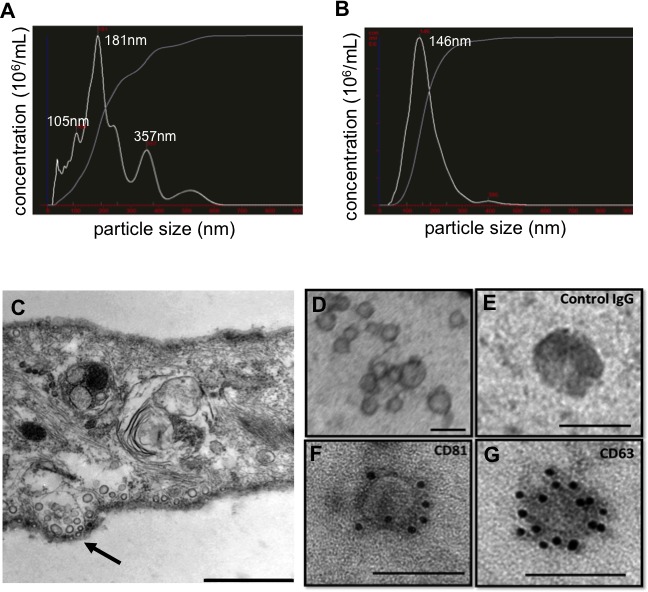
Characterization of EVs isolated from hMSCs conditioned medium (A) Particle size distribution in hMSCs conditioned media as determined by NanoSight and in (B) purified hMSCs EVs. (C) Transmission electron microphotographs of SD-MSCs, - arrow indicates vesicles at the cell membrane surface. (D) Transmission electron microphotographs of purified EVs. (E) Immuno-electron microscopy of EVs: negative IgG control. (F), CD81 detection, (G) CD63 detection. Bar represent 500 nm in C and 100 nm in D-G.

Extracellular vesicles (EVs) are the secreted small membrane vesicles (30-200 nm) that form intracellular multivesicular compartments and that are released upon fusion of these compartments with the plasma membrane. The word “extracellular vesicle” is a generic term that refers to a series of membrane-bound organelles, which are commonly distinguished by their size range. More specific nomenclature for EVs includes exosomes (40-100 nm diameter), microvesicles (50-1000 nm), and apoptotic bodies (50-5000 nm) [14]. However, there are no clear guidelines on terminologies or on different methods used for isolation and purification [15]. For the purposes of this study, extracellular vesicles (EVs) will be used for all organelles in this general category between 40-150 nm in diameter unless explicitly noted. We observed that their size varied based on cell type (Supplemental Figure S1) ranging between 100-200 nm and also varied based on the sizing technique used (Figure 1). For example when we tested EVs isolated using same technique but different sources, an osteosarcoma cell line (KHOS) and hMSCs, we have seen that the average size of purified fraction of secreted vesicles varied from 70-150 nm. Nanosight based analysis showed EVs in the sizes between 100-200 nm and electron microscopic assays demonstrated the ranges between 30-100 nm. To avoid inconsistency we have chosen to term the vesicles from SD-MSCs as extracellular vesicles (EVs), instead of exosomes. Various studies have also demonstrated a supportive role of EVs in cancer pathology, including the effects associated with cancer initiation, progression, angiogenesis, and metastasis [16-18]. Although EVs are shown to be tumor supportive and involved in transfer of various content from host cell to the recipient, none of the above studies provided a complete characterization of the EV cargo.

In this study, we isolated EVs from SD-MSCs and characterized their secreted cargo that includes small RNA, proteins, metabolites and lipids. A schematic for the data generation and analysis is presented in Supplemental Figure S2. We found that hMSCs-derived EVs are cell protective by transporting supportive miRNAs and promote breast tumor growth *in vivo.* Our findings provide evidence on how hMSCs support breast tumor growth in a nutrient deprived tumor core by secretion of EVs and suggest that these EVs provide novel targets for therapeutic intervention.

## RESULTS

### hMSCs Extracellular vesicles express CD81 and CD63

EVs were isolated from SD-MSCs through a series of ultracentrifugation steps of the conditioned media concentrate (as described in Materials and Methods), and the size of vesicles were analysed using NanoSight. While conditioned media contains heterogeneous population of vesicles ranging from 40-600 nm in size (Figure 1A), the purified fraction contained an enriched population of EVs with the mean diameter of 146 nm (Figure 1B). A series of experiments were performed to confirm the origins and morphology of the EVs. First, transmission electron microscopic (TEM) assays of serum deprived hMSCs showed the formation and secretion of microvesicular bodies that are released into the extracellular environment. TEM pictures of isolated EVs showed that the sizes varied from 30-100 nm (Figure 1C and 1D). Next, we used immunogold labelled antibodies to confirm the identity of EVs and found that purified EVs expressed specific markers for exosomes, CD81 and CD63 (Figure 1E, 1F and 1G).

### EV cargo includes anti-apoptotic proteins, bioactive lipids and metabolites

To identify and characterize the proteins, metabolites including lipids, and small molecules that may play roles in tumor cell protective function, we performed -omics based assays. The extracted peptides from EVs were analysed by LC-MS/MS for proteomics. Protein database searching and matching of the LC-MS data resulted in identifying a total of 281 proteins between the two donor hMSCs and both the donors shared 156 proteins. We compared the identified proteins with the ExoCarta database (exocarta.org) of exosomal proteins and observed that 35% of these shared proteins are found to be present within the top 25 of human originated exosomal/EVs proteins (data not shown). Furthermore, we classified the identified proteins using gene ontology (GO) based on biological process, molecular process and cellular function. The EV proteins are mainly involved in binding, rolling and intra/extravagination on cellular membrane (Table S1). Cellular components of the identified proteins revealed that the localisation of proteins is minimal in cell surface and inside nucleus (Table S1).

**Figure 2:**
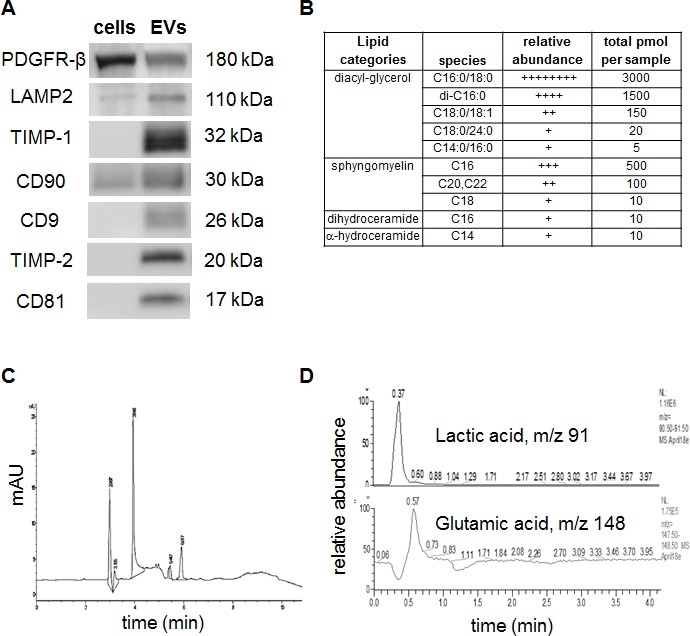
Detection of specific proteins and small molecule metabolites in EVs (A) Western blot assays to confirm the presence of exosome/EV specific markers. (B) Relative and absolute abundance of lipid species in the EVs. (C) Electropherogram obtained from separating EV lysate by free zone capillary electrophoresis with UV detection, and (D) HPLC-MS detection of lactic acid and glutamic acid in the EV lysate.

Western blot assays confirmed the expression of PDGFR-β, LAMP2, TIMP-1, TIMP-2, CD90, CD9, and CD81 among other proteins detected in proteomics analysis (Figure 2A). The relative expression of proteins in whole cells and EVs showed that LAMP2 and CD90 were enriched in EVs, whereas PDGFR-β is highly expressed in cells. Expression of CD9 and CD81, exosomal markers were expressed only in hMSCs derived extracellular vesicles. Additionally, the metallopeptidase inhibitors TIMP-1 and TIMP-2 were expressed only in EVs but not in cells.

Previous studies have shown that EV cargo is harboured with specific types of proteins, miRNAs, lipids, and mRNA [19, 20]. The lipid composition of extracellular vesicles is understudied and especially, lipidomics of EVs from hMSCs is not performed. For partial characterization of the lipid composition the isolated EVs were tested for the presence of - Sphingoid bases (sphingosine, dihydro-sphingosine), Sphingoid base-1- phosphates (S1P, dhS1P), ceramide (Cn-Cers), dihydro-ceramide, alpha-hydroxy-ceramide, diacyl-glycerol (DAGs), sphingomyelin (SM), dihydro-SM, hexosylceramide (glucosyl- and galactosyl-ceramide), lactosyl-ceramide and ceramide 1- phosphate molecular species, separated glucosyl and galactosyl ceramide species. The data revealed high abundance of diacyl-glycerol and sphingomyelin, whereas dihydro ceramide and α-hydro ceramide were seen in trace amounts (Figure 2B).

Next for metabolomics assays to characterize the small molecule or metabolite composition in the EVs, we used CE-UV and HPLC-MS/MS studies. EVs sample (5 μg protein) was lysed with methanol and the lysate was analyzed by CE-UV and HPLC-MS/MS. A typical CE-UV electropherogram obtained from the sample is shown in Figure 2C. Six peaks seen in the electropherogram indicate that they are small molecules (FW < ~500). The results indicate that small molecule compounds occur in EVs. The chromatograms obtained from HPLC-MS analysis the same sample are represented in Figure 2D. Since the levels of EVs compounds were very low, selected ion monitoring (SIM) chromatograms are shown. In the sample analyzed, lactic acid, glutamic acid, showed detectable levels of expression, while the levels of AMP, GSH, cystine, and cysteine were below the limits of detection of the present assay.

### hMSC-EVs cargo includes miRNA and lncRNA

**Figure 3:**
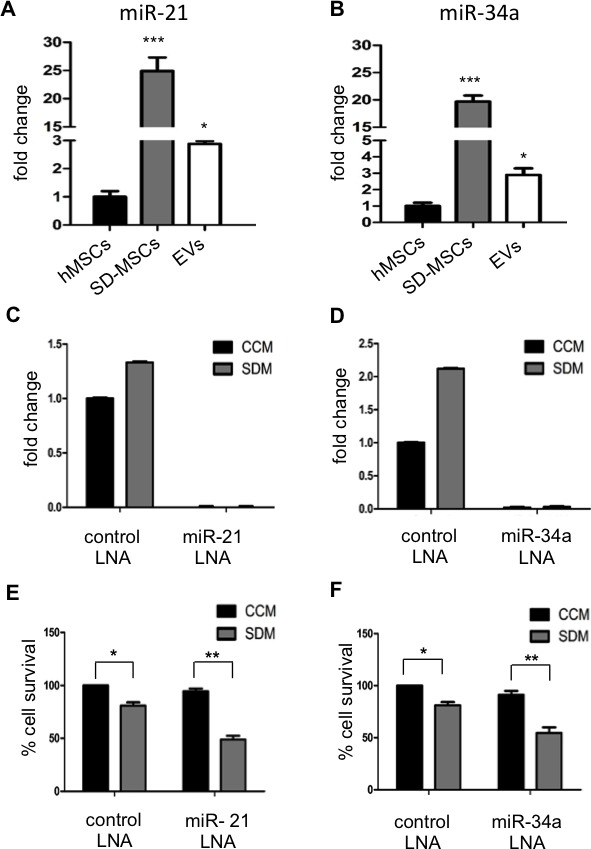
miR-21 and miR-34a are expressed in EVs (A) Relative levels of miR-21 and (B) miR-34a in normal hMSCs, SD-MSCs and SD-MSC EVs. Values were normalized to 5S RNA levels and subsequently to miRNA levels of hMSCs grown in complete conditioned media. The average of three replicates is displayed (*P < 0.05; ***P<0.001; n=3). Inhibition of miR-21 and miR-34a decreases cell survival. (C) Relative levels of miR-21 and (D) miR-34a in hMSCs (CCM) and SD-MSCs (SDM) after transfection with corresponding LNA inhibitors. Values are normalized to 5s RNA levels and subsequently to miRNA levels of hMSCs grown in complete conditioned media. The average of three replicates is displayed. (E, F) Celigo cell survival assays after treatment with LNA inhibitors (*P < 0.032; **P<0.012; n=3).

Next the nucleic acid cargo of EVs was explored using next generation sequencing for small RNA. EVs isolated from two donors were subjected to sequencing as described in methods section. The data analysis to choose for microRNA and lncRNA with known role in cell cycle regulation was performed. A schematic for data analysis is shown in Supplemental Figure S3B. The data obtained from deep sequencing data analysis, two microRNAs and two lncRNAs were chosen. miR-21 and miR-34a from miRNA group and lncRNAs 7SK and Y1 were chosen for further investigation to test our hypothesis that tumor supportive EVs includes a cargo that is anti-apoptotic. miRNAs– 21 and 34a have been demonstrated to be involved in cell survival and proliferation [21-23]. To identify the expression levels of miR-21 and miR-34a, we performed real time PCR with total RNA isolated from hMSCs, SD-MSCs and EVs. The specificity of primers for these miRNAs was confirmed by dissociation curve (Supplemental Figure S4A). We found that miR-21 was expressed ≈25 fold in SD-MSCs and ≈2-3 fold in EVs when compared to hMSCs (Figure 3A). The expression of miR-34a was ≈20 fold in SD-MSCs and ≈3 fold in EVs (Figure 3B). Furthermore, we have tested the expression of human lnc-Y1 and lnc-7SK and observed an enrichment of 2-fold in EVs compared to hMSCs (Supplemental Figure S4B). These results suggest that stress induced by serum-deprivation resulted in upregulation of miRNAs and lncRNA involved in cell survival, thereby preventing the cells from apoptosis. Increased inclusion of protective miRNAs in EVs suggests the highly regulated miRNA processing to trigger and enable protective pathways in recipient cells.

### Inhibition of miRNAs-21, and 34a induces cell death in SD-MSCs

To investigate the specific and direct role of miR-21 and miR-34a in cell survival of SD-MSCs, we performed inhibitor studies using locked nucleic acids (LNAs) specific to the miRNAs. hMSCs transfected with LNAs for their respective miRNAs were grown either in complete conditioned media (CCM) or serum deprived media (SDM) and total RNA was isolated from transfected cells with LNA control or specific LNA were analysed by real time PCR. Efficient silencing of miRNA was observed in the transfected cells (Figure 3C, 3D). The amplified product of PCR was run on agarose gel to confirm the miRNAs silencing (Supplementary Figure S5A). Interestingly, in addition to SD-MSCs, inhibition of miRNAs 21 and 34a resulted in decreased survival of cells grown in CCM. Inhibition of miRNAs-21 and 34a resulted in decreased cell survival of hMSCs in SDM representing their importance in inducing protective pathways under stress conditions in the form of serum deprivation (Figure 3E, 3F, and Supplemental Figure S5B). Similar results were observed in hMSCs from different donors (data not shown). Taken together, EVs from hMSCs that carry miRNAs-21 and 34a are protective in function for hMSCs under serum-deprived conditions.

### EVs secreted by hMSCs increase survival of cancer cells under stress

**Figure 4:**
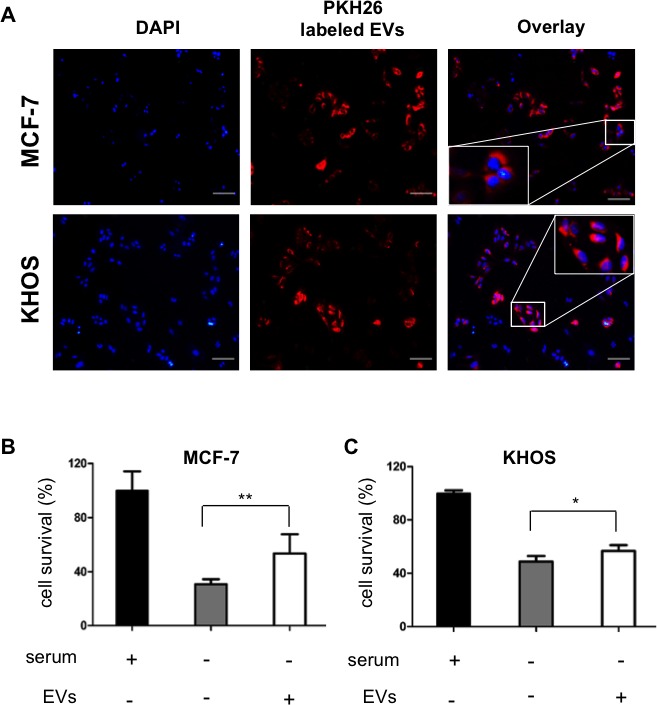
hMSCs derived EVs are internalize by cancer cells and support cell survival (A) MCF-7 breast cancer cells and KHOS osteosarcoma cells incubated with PKH26 labeled SD-MSCs derived EVs for 3 h. EVs have been internalized by cancer cells. Bar represents 100 μm. Insets shows the magnified image. (B) MCF-7 and (C) KHOS cells were cultured in either serum containing media or serum deprived media or serum deprived media supplemented with EVs. The cell survival is quantified by Cyquant DNA quantification method (*P < 0.05; **P<0.018; n=3).

To study the role of EVs in cell survival, EVs derived from SD-MSCs were labelled with cell membrane marker PKH26 (red fluorescence) and incubated with breast cancer (MCF-7) or osteosarcoma (KHOS) cells for 3 h and viewed under fluorescence microscope. The cells were scored for internalized EVs, and over 50% of tumor cells (Figure 4A), as shown by red fluorescence inside cells. Next, the functional anti-apoptotic fraction of the SD-MSCs group was quantified using a well-established model, where serum deprivation induces apoptosis in the cells [9]. MCF-7 and KHOS cell lines were grown in complete medium replaced with serum free media for 24 hr supplemented with EVs or not supplemented. Serum deprivation induced apoptosis in both MCF-7 (Figure 4B) and KHOS (Figure 4C) cells, indicated by a significant decrease in the total number of surviving cells. However, in the presence of EVs over 15% increase in cell survival is observed. Specifically, MCF-7 exhibited better response to the presence of EVs (25%) compared to KHOS (15%) (Figure 4B, 4C). These results demonstrate that extracellular vesicles derived from hMSCs exhibit anti-apoptotic function or cell protective function.

### EVs support breast tumor growth *in vivo*

**Figure 5:**
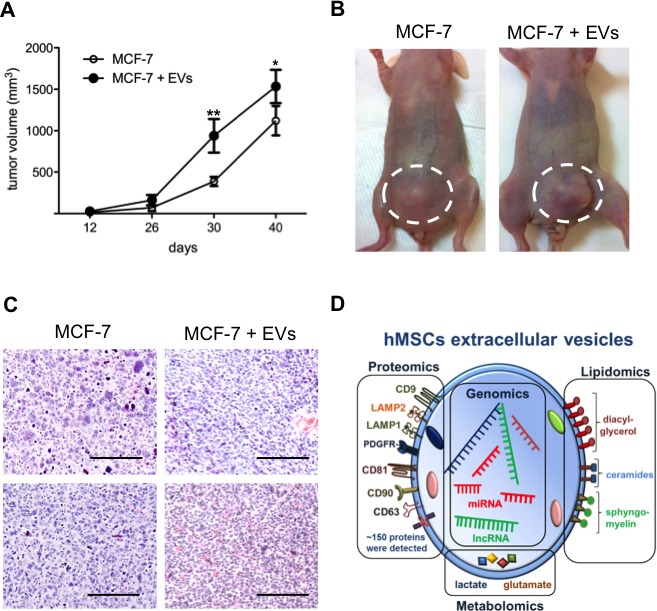
EVs enhance breast tumor growth (A) Xenograft assays of MCF-7 cells with or without EVs. The tumor sizes of MCF-7 + EVs were significantly higher than MCF-7 alone (*P < 0.05; **P<0.01; n=4). (B) Representative photos of tumors from each cohort orthotopically injected MCF-7 alone and MCF-7 and EVs, showing difference in tumor size (highlighted by dashed circles). (C) Hematoxylin and eosin stained sections showed tumors from MCF-7 + EVs have higher angiogenesis when compared to MCF-7 alone. (D) Schematic representation of hMSCs extracellular vesicles major components.

Cancer-derived EVs and exosomes in tumor environment influence cancer progression (reviewed in [24]). Next, we investigated the potential of hMSCs-derived EVs on the growth of breast tumors in immunodeficient mouse model. Equal numbers of MCF-7 cells (1 × 10^6^) were injected in mammary fat pads with matrigel in two groups of mice. One group received the cells alone and the other with 20 μg EVs from SD-MSCs. Detectable masses of tumors were seen in both the groups from 2 weeks (Figure 5A). Larger tumors are seen in mice group, which received MCF-7 cells along with EVs when compared to mice received MCF-7 (Figure 5A, 5B) cells alone. These results indicate that hMSCs derived EVs indeed support breast tumor growth *in vivo*. The hematoxylin and eosin staining of the tumors demonstrated that the tumors co-injected with EVs exhibited higher angiogenesis (Figure 5C).

## DISCUSSION

Over the past several years, a significant amount of research has emerged documenting a role for hMSCs in accelerating tumor growth and metastasis [2, 24]. Given this complex interplay between hMSCs and tumor cells intercellular communication; the goal of this study was to assess the role of EV transfer from hMSCs to tumor cells. Several s rowth factors to enhance cancer cell growth [29, 30], alters the cancer cell metabolism [31] and increase their tumorigenic ability [32]. Furthermore, CAFs increase the invasiveness of the cancer cells.

Previous findings from our laboratory [9, 10, 33, 34] demonstrated that early passage hMSCs when subjected to serum deprivation selects an active and functional subpopulation of very early progenitor cells. These SD-MSCs remains undifferentiated but retains their differentiation potential into osteoblasts, chondrocytes, and adipocytes [10]. Furthermore, hMSCs utilize autophagy pathways to provide stromal support in a nutrient deprived and poorly vascularized core of solid tumors. In addition, experiments with hMSC secretome in tumor progression, demonstrated that epigenetic changes under stress direct expression of tumor supportive growth factors [9, 34, 35]. Initial secretome characterization studies performed on SD-MSCs showed that they secrete tumor supportive factors such as VEGF-A, HGF, IGF-1 [35]. However those studies have not completely expounded on the molecular composition and mechanisms of the protective function of secretome. We have chosen to use SD-MSCs for studies related to EVs in tumor microenvironment because they offer several advantages: a) EV isolation and characterization will be true to the cell type as issues related to serum contamination are abrogated, b) the phenotype of SD-MSCs is closer to the stromal cell phenotype offering the functional advantage.

The four known components that have a potential role in EV mediated cell-cell communication are small RNA, proteins, lipids and metabolites. We characterized important components of the EVs using comprehensive proteomic, genomic, lipidomic and metabolomics assays. This is the first report on complete biochemical and molecular characterization of EVs isolated from hMSCs.

It has become imperative to support the studies using EVs to include in-process data on their preparation because of the variability associated with various purification techniques [14]. Figure 1 demonstrates that ultracentrifugation purification assays have concentrated 70-150 nm extracellular vesicles. The purified extracellular vesicles are double membrane and immuno-electron-microscopic assays confirmed that EVs express CD81 and CD63, known exosome/microvesicle markers. A discrepancy is observed between two commonly used methods for EV size determination, Nanosight and electron microscopic assays; while the former identified the size to range between 100-200 nm, the electron microscopic images show particles between 30-100 nm. The inconsistency between techniques indicate a need for further investigation into developing a dependable assays for EV size determination. Based on the size determination and marker expression it is safe to assume that we are dealing with exosomes-type vesicles. However, we believe that defining a secretory vesicle based on size could be rather misleading especially in situations where the functional properties of the vesicles may be shared among various fractions of the secretome. Our primary goal of studying EVs to understand their biological roles, therefore we prefer to use the term EVs to define the secreted vesicles, which fall in the size range of 40-150 nm.

Consistent with observations from other groups in different models [36], we demonstrated that the protein composition of EVs is tumor supportive and more than 30% of proteins being anti-apoptotic and cell proliferative. As a proof of concept we have confirmed expression of 7 proteins; two proteins confirm the origin of the hMSCs EVs (CD90 and CD81) [37] and five known proteins associated with tumor proliferation and anti-metastasis [38-42]. Proteomics data supports the notion that the protein sorting in the EVs is not a random event: a) the constancy between the proteomic assays on EVs preparations from two different donors. b) The total number of proteins present in the preparation is less than 300, which are well within the range of number of proteins to be packaged into a less than 200 nm vesicle. This data further supports our conclusion that the EVs used in this study are homogenous population with limited contamination from commonly co-purified apoptotic blebs [43] and serum components that are rich in proteins. The variability of protein expression in EVs that are not seen in whole cell western could be explained by two possible scenarios: (a) a well regulated packaging of EVs with proteins that may not be highly expressed in cells (b) the western blot is normalized to the same amount of total protein, the data seen is function of ratio of specific protein to total number of proteins.

Previous studies have suggested that lipid rafts may play a role in EV biogenesis and structure. The lipid metabolism studies in cancer has shown that alterations can affect the availability of structural lipids for the synthesis of membranes, the synthesis and degradation of lipids that contribute to energy homeostasis and the abundance of lipids with signaling functions. We tested the presence of bioactive lipids that are characteristic of EVs, the production of which is regulated by the ceramide pathway. HPLC/MS-MS lipid profiling demonstrated typical raft components were in hMSC-EVs. Relative abundance of diacyl-glycerol, sphingomyelin and ceramides further suggest the origins of the EVs through ceramide pathway as shown previously [44, 45 ]. The presence of sphingomyelins and diacylglyceraol data further supports the hypothesis that lipid raft composition in EVs adds to the functional role of hMSC-EVs to support tumor growth.

Similar to protein and nucleic acid, the metabolism is altered in tumors. Small molecule metabolites play a significant role in the breast tumor expansion. Warburg-like metabolism is influenced by changes in stromal-epithelial cell interactions, including metabolism related genes and epigenome [46]. Preliminary assays for metabolites has shown that EVs from hMSCs contain glutamic acid and lactic acid both of which are associated with tumor proliferation [47]. Glutamine is a known amino acid that traffics both carbon and nitrogen and provides precursors for basically all of the major macromolecular classes. The presence of lactic acid in the tumor microenvironment is associated with the increased ability of tumor cells to survive hypoxic and nutrient deprived core [48]. The low pH generated through lactic acid secretion through modified glucose/glutamine metabolism is attributed to cancers escape from immune surveillance. This data further supports our hypothesis that hMSCs provide an ideal tumor supportive environment, which includes lactic acid secretion.

To study the small nucleic acid components we performed deep sequencing assays and PCR array for known miRNA and lncRNA. A series of bioinformatics analysis revealed miRNA transported by EVs which are involved in functions like cell death, proliferation and survival. As a proof of concept, we performed over expression knock out assays for miRNAs-21 and 34a, which supported our hypothesis [49]. Taken together, this genomic data provides a proof of concept that EVs from stressed hMSCs act as carriers that transport tumor supportive miRNA and lncRNA. MicroRNA-21 is shown to stimulate proliferation in renal cell carcinoma and breast carcinoma [50, 51]. MicroRNA-34a has been shown as a tumor supportive and inhibitory microRNA, putting it in the group of microRNA that exert contradictory functions [52, 53]. The anti-proliferative (or tumor suppressive) effect of miR-34a is associated with its role in targeting transcription factor E2F3, however the cancers that have high expression of miR-34a were shown to have low levels of E2F3 [52]. Taken together, our data demonstrates that miRNAs-21 and 34a play a cell proliferative role in MCF-7 cells. As seen *in vitro*, pretreatment of EVs inhibited cell death when MCF-7 and KHOS cells were treated with serum free media. In addition, the *in vivo* assays verified that tumor supportive properties of hMSC-EVs. These results suggest that the transfer of small amounts of exogenous small RNA, proteins and lipids may aid in tumor progression. Functional assays to demonstrate that the EVs from hMSCs are breast tumor supportive corroborate our previous observations [9].

Metastatic breast cancers at stage 4 have a mortality rate of 85% in five years, while stage one is 15% [54]. Therefore, there is an urgent need to develop diagnostics for early identification of metastatic phenotype. While several groups are interested in studying the exosomes secreted by the tumor cells, this is the first comprehensive study to understand exosomes or EVs secreted by stromal cells. The integral role of stroma in tumor growth, dormancy and metastasis is well studied. In addition, the stromal components undergo changes in the solid tumor, understanding such changes would potentially provide a non-invasive diagnostic method for metastatic breast cancers. Our model mimics such changes that stromal cells would undergo with nutrient deprivation in a primary tumor. Therefore, further studies to explore the stromal EVs cargo would shed light on novel diagnostics for metastatic breast cancers.

In conclusion, this report offers a comprehensive analysis of extracellular vesicular cargo that sheds light on the cell communication in the tumor microenvironment. In this study, we show that EVs from stressed SD-MSCs act as rafts to carry tumor supportive proteins, miRNA, lipids and metabolites. Further studies are warranted to determine which of these components trigger the right molecular mechanisms in the tumor cells.

## MATERIALS AND METHODS

### Cell culture

hMSCs from bone marrow aspirates were provided by the Texas A&M Health Science Center College of Medicine Institute for Regenerative Medicine at Scott & White through a grant from NCRR of the NIH, Grant # P40RR017447. The cells were obtained as frozen vials of passage 1 cells that were shown to be multipotent for differentiation. The cells were negative for hematopoietic markers (CD34, CD36, CD117, and CD45), and positive for CD29 (95%), CD44 (>93%), CD49c (99%), CD49f (>70%), CD59 (>99%), CD90 (>99%), CD105 (>99%) and CD166 (>99%). All cultures were cultured as described previously [9, 55]. MCF-7 and KHOS cell lines were obtained from ATCC and grown according to the recommended culture conditions. MCF-7 cells expressing GFP and luciferase used for *in vivo* experiments were generated previously [56].

### Extracellular vesicles (EVs) isolation and characterization

EVs were isolated using a modified protocol described by Thery et al. [20]. Briefly, hMSCs were cultured in CCM until they were 80% confluent in two-layered cell factories (Nunc) (30 × 10^6^ cells). CCM was replaced with SDM and cells were grown under these serum-deprived conditions for a period up to 15 to 30 days. Conditioned media of 250 ml was collected every 3 days, centrifuged at 500 g for 10 min to eliminate cell debris. The supernatant was concentrated using a positive pressure concentrator (Amicon 8400) with 1 kDa ultra-filtration discs (Millipore) to a final volume of 5 ml and ultra-centrifuged at 15,000g for 1 h at 4°C to remove large vesicles, the supernatant which contain the EV fraction was further subjected to ultracentrifugation at 110,000 g for 18 h at 4°C. EV pellets were washed with PBS and ultracentrifuged at 110,000 g for 18 h at 4°C and pellets were resuspended in 100 μl of PBS and aliquots were stored at −80°C. EVs, were characterized using NanoSight LM10 system (NanoSight Ltd, Amesbury, UK) and assayed with NTA software version 2.3 as described elsewhere [57].

### Electron microscopy

Purified EVs were fixed in 4% paraformaldehyde (PFA) and deposited on Formvar carbon coated TEM grid in dry environment for 20 min. After EVs adsorption, grid was washed two times in PBS followed by three times in PBS/50 mM glycine for 3 min each. Then grid was transferred to a drop of blocking buffer (5% BSA in PBS) for 10 min. EVs were immunolabeled with mouse anti-human CD-63 (Clone H5C6, BD Biosciences, San Jose, CA) and mouse anti-human CD-81 (Clone 1D6, Leica Microsystems, Buffalo Grove, IL) separately for 30 min. Antibody dilutions tested were 5 μg/ml as mentioned in the protocol by Thery et al. [20]. Control grids were incubated with mouse IgG1 (Clone MOPC-21, BD Biosciences, San Jose, CA). The grids were washed, treated with secondary antibody, stained with uranyl acetate and embedded in 4% uranyl acetate and 2% methylcellulose as mentioned published [20]. The specimens were examined under acceleration voltage of 200 kV under a JEOL 2011 transmission electron microscope.

### EVs PKH26 labeling

EVs were labeled using the PKH26 red fluorescence cell linker kit (Sigma, St. Louis, MO) according to manufacturer's instructions, EVs were washed four times with PBS. Labeled EVs were incubated with cells for 3 h on coverslips in a 24-well plate and viewed under Nikon Eclipse 80i fluorescence microscope and analyzed with NIS-elements BR software version 4.0.

### Proteomics

Sample Preparation: To prepare mass spectrometry compatible peptides from EVs pelleted proteins, disulfide bonds were reduced with 20 mM dithiothreitol (DTT from Sigma-Aldrich, Lyon, France) for 30 min at 56°C, free thiols were then alkylated with 50 mM of chloroacetamide for 30 min at room temperature. The protein precipitate was resuspended in digestion buffer containing 50 mM ammonium bicarbonate (Sigma Aldrich, Lyon, France), 1% RapiGest (Waters, En Yvelines Cedex, France) and 0.2 μg of sequencing grade trypsin (Promega, Lyon, France) and digested 3 h at 37°C under frequent agitation. The reaction was stopped with 1 μL of 10% TFA (Fluka, Lyon, France). Resulting peptides were collected for subsequent analysis.

Nano Liquid Chromatography with Tandem Mass Spectrometry Analysis (LC-MS/MS):

Peptide concentration and separation was performed using a nanoflow liquid chromatographic system (Ultimate 3000 RSLC from Dionex) coupled to a hybrid mass spectrometer (Linear Trap Quadrupole-Orbitrap Velos from Thermo Fisher Scientific) for peptide identification following manufacturer's protocol. Trap CID MS/MS was performed on multi-protonated peptides in a data dependent scheme allowing up to 10 isolations and fragmentations between each high resolution full scan MS measurement of the continuous elution process.

Protein Identification:

All LC-MS/MS results were analyzed using Proteome Discoverer 1.2 software (Thermo Fisher Scientific) in combination with Mascot MOWSE search algorithm [58] using the sequences contained in the January 2011 Swiss-prot FASTA database. Precursor mass tolerance was set to 2 ppm and fragment mass tolerance to 0.45 Da. Peptide false positive identification probability was <5%. All identified proteins from several injections were compared using the MyproMS data parser [59]. Proteins were confidently identified with at least 2 different peptides with a score of identification ≥20.

### Metabolomics

Lipids: EVs from two donors were isolated and multicomponent LC-MS/MS and/or SFC/MS/MS analysis was performed at Medical university of South Carolina lipidomics and pathobiology core. The EVs were tested for the presence of - Sphingoid bases (sphingosine, dihydro-sphingosine), Sphingoid base-1- phosphates (S1P, dhS1P), ceramide (Cn-Cers), dihydro-ceramide, alpha-hydroxy-ceramide, diacyl-glycerol (DAGs), sphingomyelin (SM), dihydro-SM, hexosylceramide (glucosyl- and galactosyl-ceramide), lactosyl-ceramide and ceramide 1-phosphate molecular species, separated glucosyl and galactosyl–ceramide species using methods (LC-MS/MS and/or SFC/MS/MS) described in [60, 61].

### Small molecule characterization

EVs sample was mixed with 200 μl methanol and sonicated for 30 sec. The lysate was analyzed by CE-UV and HPLC-MS/MS. CE conditions: column; 75 μm ID /370 μm OD x 50 cm long fused-silica capillary; sample injection, pressure at 50 mbar for 10 sec; CE running buffer, 20 mM Tris-HCl buffer (pH 8.2); CE voltage, positive 25.0 kV; column temperature, 20°C. CE-UV analysis. An Agilent CE instrument (7100 Model) was used.

### HPLC-MS analysis

The system consisted of a Surveyor HPLC and a TSQ triple quadrupole mass spectrometer equipped with a heated ESI source (Thermo Scientific, San Jose, CA, USA). Xcalibur software was used for data acquisition and process. A C_18_ reversed-phase column (Ascentis® 3 μm particle size, 10 cm × 2.1 mm, Sigma-Aldrich Chemicals, St, Louis, USA) was used for separation. MeOH/water mixture (50/50, v/v) containing 0.1% formic acid was used as the mobile phase at a flow rate of 0.200 ml/min. Sample injection volume was 5 μl. The MS detector was operated in the positive ion mode with the following settings: spray voltage 3 kV, vaporization temperature 270°C, capillary temperature 300°C, sheath gas pressure 35 (arb), auxiliary gas pressure 10 (arb), tube lens voltage of 150 V, and capillary voltage of 35V.

### Genomics

Next generation sequencing: The small RNAs isolated from the EVs that are secreted from hMSCs were processed to generate a cDNA library, which was then used for deep sequencing. Sequencing of small RNAs from hMSCs EVs resulted in ≈16 million raw reads (Supplementary Figure S3A), out of which ≈13 million were mappable reads. The mappable reads were grouped into several groups. Group 1a included miRNAs from human miRBase of specific species and this group was considered for further evaluation. First, miRNAs from group 1a were sorted based on the copy number, function and *z* score. A schematic for miRNA analysis is shown in Supplemental Figure S3B. Overall, the miRNAs from group 1a were divided into 6 groups based on their functions. Similarly long non-coding RNAs (lncRNAs) were sorted based on 8 mers sequence after eliminating duplicates and finally 4 lncRNAs were shortlisted (Supplemental Figure S3B). A miRNA microarray analysis was first performed on 585 known miRNAs sequences and we identified 134 miRNAs, which are negative regulators of apoptosis. Data crossover between the next generation sequencing and miRNA microarray confirmed that at least 10 miRNAs identified as involved in cell death, survival and proliferation of cells. For proof of concept, we selected miR-21 and miR-34a that are known to be involved in cell survival and proliferation.

### Western blots

EVs secreted by SD-MSCs from two different human donors (days 36-40) were lysed in RIPA buffer (Santa Cruz, Dallas, TX) and protein contents estimated using microBCA assay (Pierce, Rockford, IL) and western blot assays were performed us 10 μg of proteins. Antibodies: PDGFR-β (Santa Cruz, cat# sc-432, 1:200), LAMP2 (Thermo Scientific, cat# MA1-20798, 1:200), TIMP-1 (Chemicon, cat#AB800, 1:1000), CD90 (BD Pharmingen, cat#555596, 1:200), TIMP-2 (Chemicon, cat#AB801, 1:1000), CD9 (AbCam cat#ab2215) and CD81 (AbCam cat#ab79559).

### Locked nucleic acid transfection

Cells were transfected with locked nucleic acids (LNAs) (Exiqon, Woburn, MA) using HappyFect reagent (Tecrea, London, UK) according to the manufacturers protocols. LNAs are used at a concentration of 10 pmol/well, 20 pmol/well, and 50 pmol/well in 24, 12, and 6-well plates, respectively. Sequences of miRCURY LNA inhibitors are:

hsa-miR-21 5′-3′ CAACATCAGTCTGATAAGCT/36-FAM;

hsa-miR-34a 5′-3′ ACAACCAGCTAAGACACTGCC/36-FAM.

### Real Time RT-PCR analysis

For miRNA PCR arrays, total RNA was extracted from hMSCs or SD-MSCs using the RNeasy Mini Kit (Qiagen, Valencia, CA), and 500 ng of RNA was converted into cDNA with the RT2 First Strand Kit (SuperArray Bioscience, Frederick, MD). Real-time PCR was performed using the RT^2^ miRNA PCR array with the RT^2^ SYBR Green Master Mix (both SuperArray Bioscience, Frederick, MD) according to the manufacturer's protocol. PCR arrays were run with an ABI PRISM® 7900HT Sequence Detection System, (Applied Biosystems; Foster City CA) using the SDS 2.2 program.

For miR-21 and 34a studies, total RNA was isolated from hMSCs, SD-MSCs and EVs using mirVana kit (Ambion, Grand Island, NY) according to manufacturer's protocol. cDNA was established from 500 ng of total RNA using miScript II RT Kit (Qiagen, Valencia, CA), Real-time PCR was performed with cDNA in triplicates using miScript SYBR Green PCR Kit (Qiagen, Valencia, CA) in CFX96 Real-Time PCR system (Bio-Rad, Hercules, CA). miRNA are amplified using the following primers, hsa-miR-21 5′-TAGCTTATCAGACTGATGTTGA-3′, hsa-miR-34a 5′-TGGCAGTGTCTTAGCTGGTTGT-3′, 5sRNA 5′-TACGGCCATACCACCCTGAA-3′ and 3′-GCGGTCTCCCATCCAAGTAC-5′. Anti-sense primers were the universal primer set available in the kit.

### Qualitative analysis of cell survival using Celigo

Cells were transfected in 24 well plates in duplicates with LNAs using appropriate controls and analyzed for cell survival after 36 h using automatic cell analyzer Celigo (Cyntellect, San Diego, CA). Cells were fixed with 4% PFA for 10 min and after two washes stained with 0.5% v/v Hoechst 33342 (ImmunoChemistry Technologies, Bloomington, MN) for 10 min. After washing away the excess stain, the plate was scanned using Target 1 pre-program in Celigo. For analysis, cell area parameter was set at 120-10000 pixels to exclude cell debris and all the remaining parameters were default. Cell count was obtained based on the stained nuclei. Percentage cell survival was calculated after normalizing the values to respective negative controls from three individual experiments.

### Animal experiments

*NU/NU* female mice were purchased from Charles River Laboratories (Wilmington, MA). All animal studies were conducted in accordance with NIH animal use guidelines and a protocol approved by UMMC Animal Care Committee. Mice were 3 to 5 weeks and were randomly divided into 2 groups (n=8). MCF-7 gfp/luc cells were trypsinized and washed twice with PBS. Tumor volumes were calculated using the formula V= (4/3)πa^2^b, where a is shorter radius in mm and b is longer radius in mm.

### Hematoxylin and Eosin staining

Breast tumors from different groups were excised from mice for H&E staining as described previously [62].

### Statistical analysis

*P* values were calculated using Student's two tailed *t* test. Differences were considered significant at *P* <0.05.

## SUPPLEMENTARY MATERIAL FIGURES AND TABLE




