# Supplementary material for: Extracellular vesicles from bone marrow mesenchymal stem/stromal cells transport tumor regulatory microRNA, proteins, and metabolites
Source: Oncotarget. 2014 Dec 31;6(7):4953–67. doi: 10.18632/oncotarget.3211 (PMC4467126; doi:10.18632/oncotarget.3211)
Supplement: Supplementary file 1 [file oncotarget-06-4953-s001.pdf]

Extracellular vesicles from bone marrow mesenchymal stem/stromal cells transport tumor regulatory microRNA, proteins, and metabolites

Supplementary Material

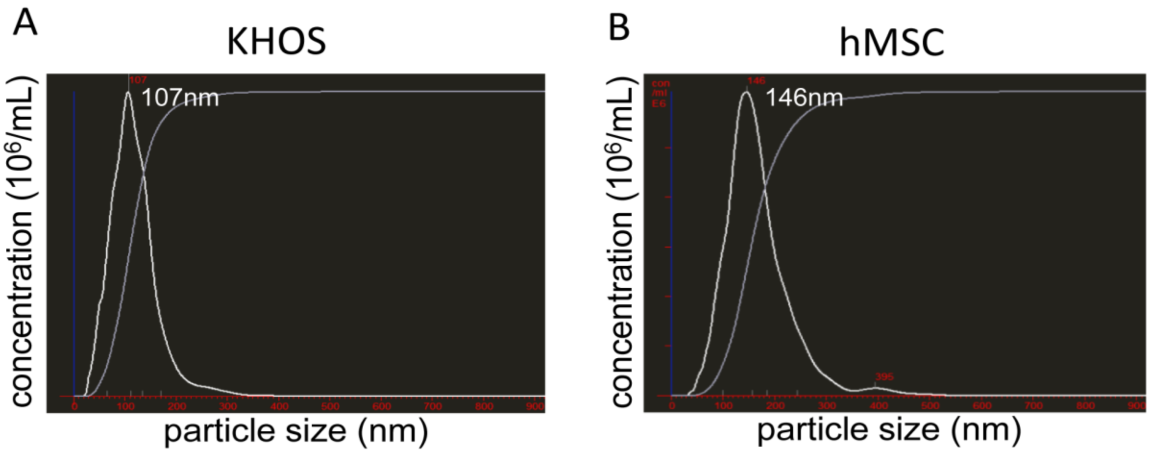

Figure S1: (A) Particle size distribution of purified KHOS EVs and (B) purified hMSCs EVs as determined by NanoSight

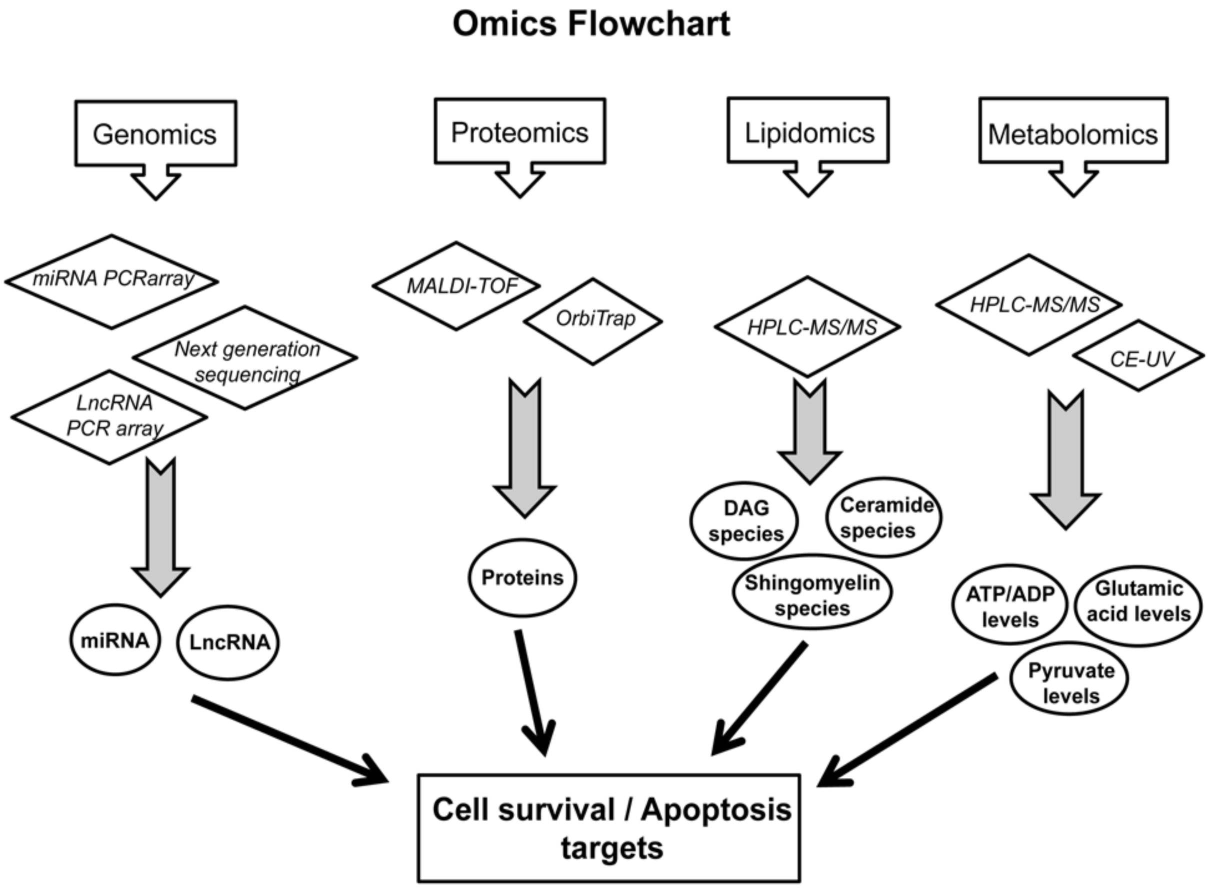

Figure S2: Flow chart of the genomics, proteomics, lipidomics and metabolomics data analysis.

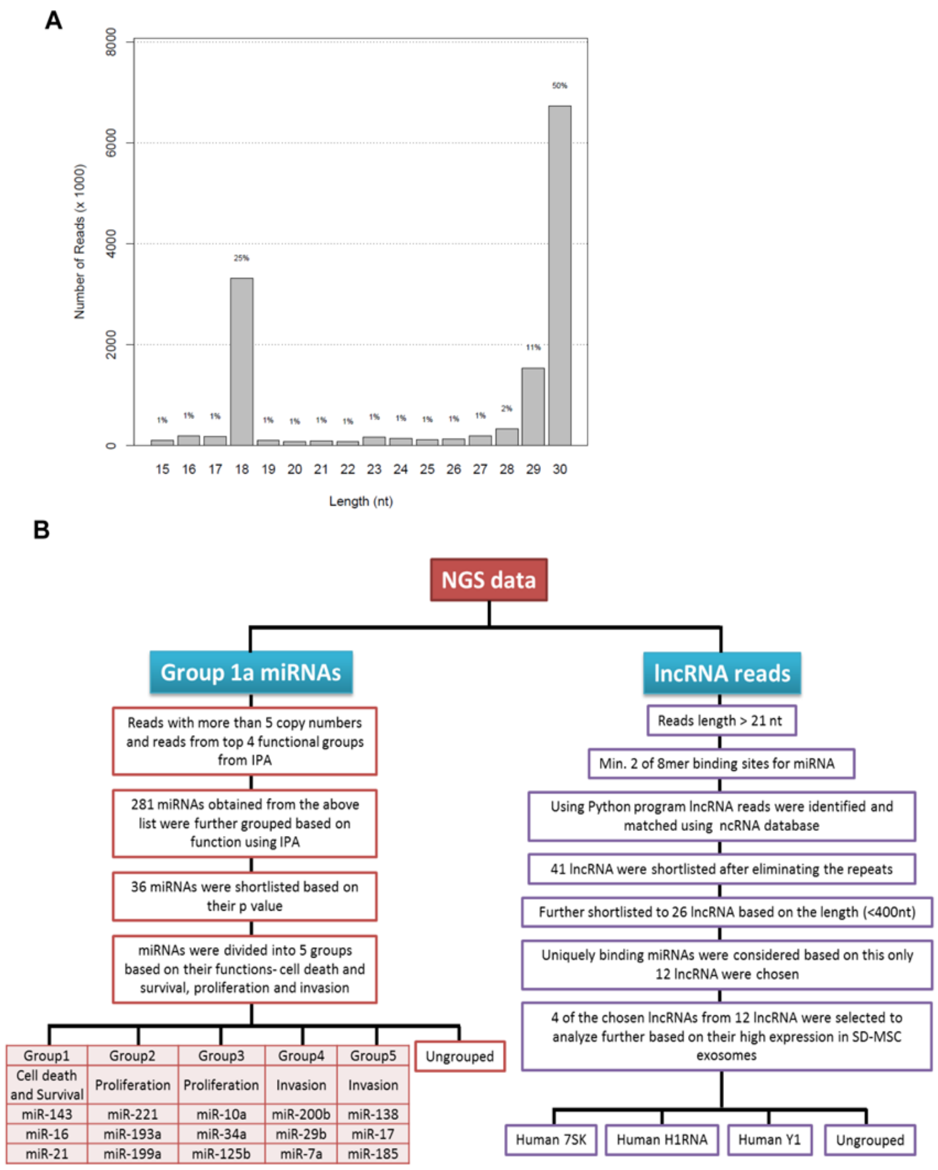

Figure S3: (A) Distribution of hMSC EVs small RNA species population (~16 million reads) sorted by nucleotides length (cutoff at 30 nts). (B) Flowchart of miRNA and lncRNA data analysis.

A

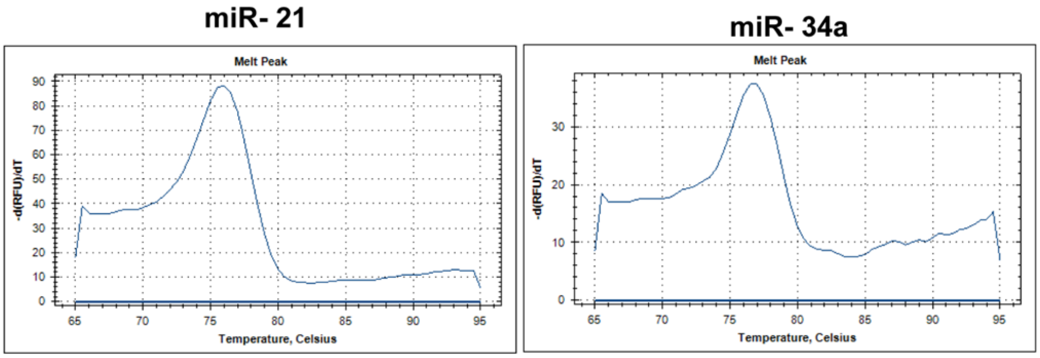

B

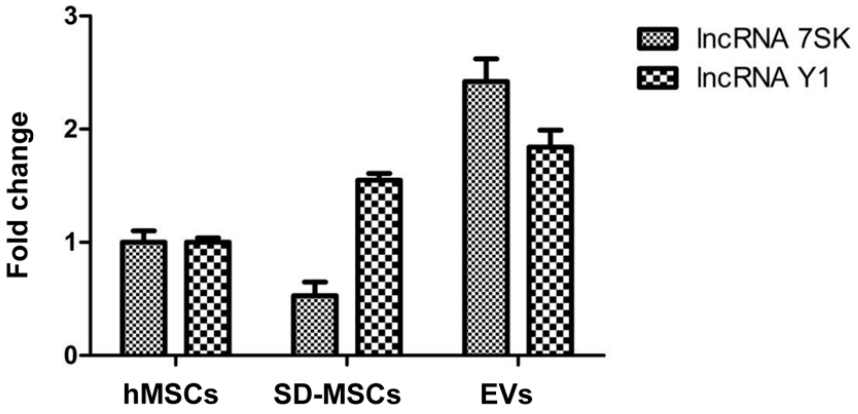

Figure S4: (A) Dissociation curves of miRNAs- 21 and -34a confirming the specificity of their respective primers. (B) Relative expression of lncRNAs 7SK and Y1 in hMSCs, SD-MSCs and SD-MSCs EVs.

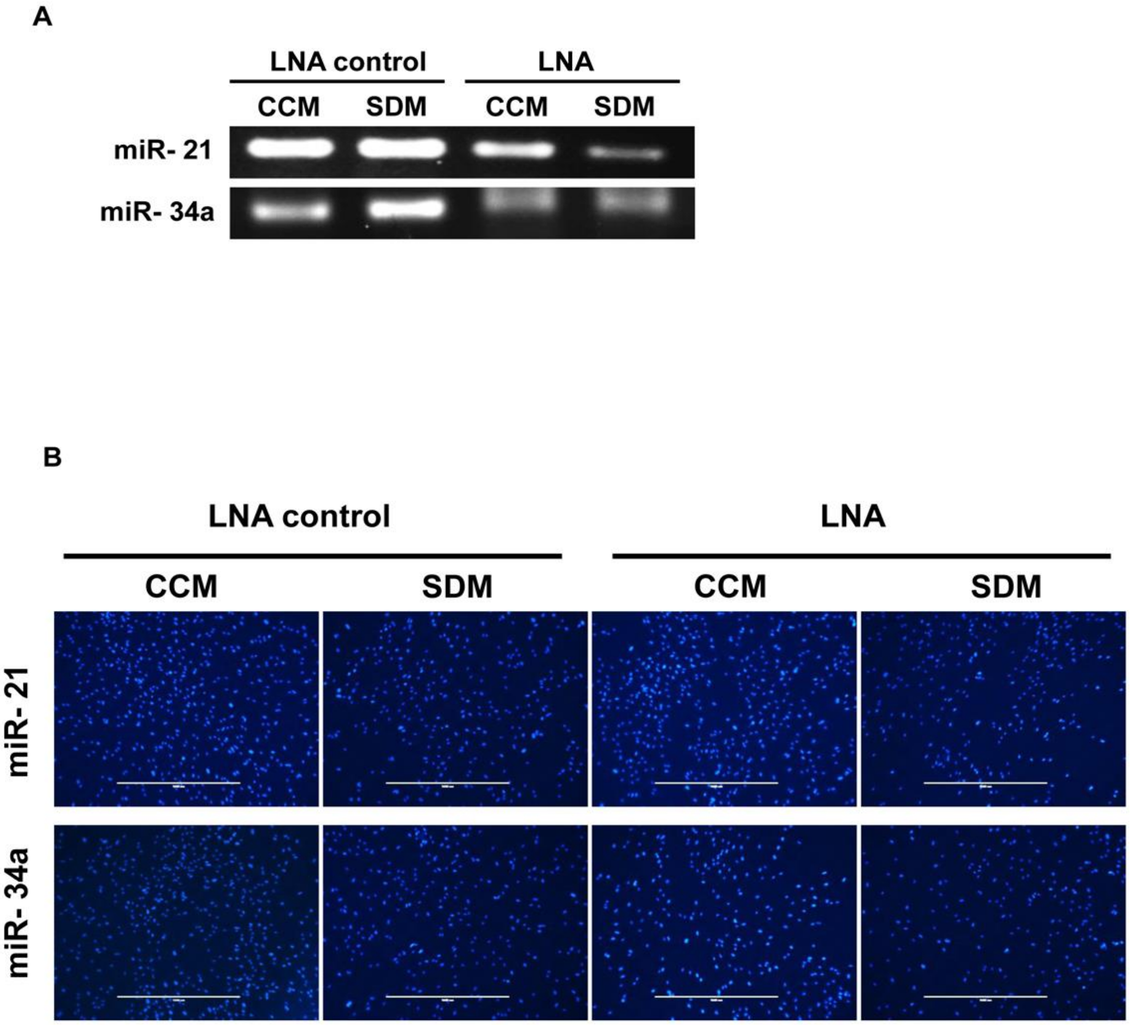

Figure S5: (A) Gel electrophoresis of miR- 21 and miR- 34a amplified product confirming silencing of respective miRNAs. (B) Representative pictures showing DAPI (blue) stained nucleus for Celigo cell survival analysis after transfections without or with miR- 21 or miR-34a LNAs inhibitors.
